# Phosphorylated neurofilament heavy chain (pNfH) concentration in cerebrospinal fluid predicts overall disease aggressiveness (D50) in amyotrophic lateral sclerosis

**DOI:** 10.3389/fnins.2025.1536818

**Published:** 2025-03-12

**Authors:** Julia Meyer, Nayana Gaur, Janina von der Gablentz, Bernd Friedrich, Annekathrin Roediger, Julian Grosskreutz, Robert Steinbach

**Affiliations:** ^1^Precision Neurology of Neuromuscular and Motor Neuron Diseases, University of Lübeck, Lübeck, Germany; ^2^Laboratory Animal Centre, Institute of Biomedicine and Translational Medicine, University of Tartu, Tartu, Estonia; ^3^Department of Neurology, Jena University Hospital, Jena, Germany; ^4^Center for Rare Diseases, University Hospital, Jena, Germany; ^5^Cluster for Precision Medicine in Inflammation, Universities of Kiel and Lübeck, Lübeck, Germany

**Keywords:** amyotrophic lateral sclerosis, neurofilament, disease aggressiveness, D50 model, ALS, progression rate, biomarkers

## Abstract

**Introduction:**

Amyotrophic lateral sclerosis (ALS) is a progressive neurodegenerative disorder, characterized by tremendous clinical heterogeneity that necessitates reliable biomarkers for the trajectory of the disease. The potential of phosphorylated Neurofilament-Heavy-chain (pNfH) measured in cerebrospinal fluid (CSF) to mirror disease progressiveness has repeatedly been suggested but is not applicable as outcome on an individual patient-level. This potential was probably obfuscated before due to imprecise clinical measures of disease progression that assumed a linear decline of motoric function over time. The primary objective was therefore to study if disease aggressiveness, as quantified via the D50 model, would reveal more stable correlations with pNfH.

**Methods:**

ELISA-quantified pNfH CSF levels of 108 patients with ALS were comparatively analyzed in relation to three different measures of disease progression speed via analyses of covariance, linear and non-linear regressions, respectively. These were (a) the D50, depicting a patient’s overall disease aggressiveness, (b) cFL, the calculated functional loss-rate as locally derived parameter of progression speed, and (c) DPR, the disease progression-rate as more commonly used linear approximation of points lost per month in the ALS functional rating scale since symptom onset.

**Results:**

All analyses of covariance showed a significant main impact of the respective disease progression-speed parameter on pNfH, independent of disease phase, presence of frontotemporal dementia, analyzing laboratory, sex or clinical onset type, while only age revealed borderline additional influence. Notably, CSF pNfH concentration was independent of how far the disease had progressed, as neither disease phase nor a direct regression with the quantified disease accumulation at the time of lumbar puncture revealed a significant correlation. However, the parameter D50 quantifying aggressiveness showed the most significant impact on pNfH-levels, as compared to the cFL and even more evident in contrast to the DPR. This superiority of D50 was confirmed in direct linear and most evident in non-linear regressions with pNfH.

**Conclusion:**

Overall disease aggressiveness in ALS, as quantified by D50, most robustly correlated with CSF pNfH-levels, independent of the time of collection during symptomatic disease. This opens perspectives to use CSF pNfH as a prognostic outcome measure for future therapeutic interventions in the sense of precision medicine.

## 1 Introduction

Amyotrophic lateral sclerosis (ALS) is a neurodegenerative disease that pre-eminently affects the motoneuronal system and has a median survival time of 3 years after symptom onset. The age and site of onset, clinical spread and rate of functional decline all vary considerably among patients (Foster and Salajegheh, 2019; Feldman et al., 2022). In addition patients are often diagnosed with delay or even misdiagnosed (Paganoni et al., 2014; Behzadi et al., 2021). This considerable variability represents a significant challenge for both clinical management and the development of new therapies. Accordingly, a major focus in the field has been the validation of reliable biomarkers that can assist with diagnoses and/or monitor disease progression (Dreger et al., 2022; Sanchez-Tejerina et al., 2023). To date, therapeutic trials applied outcome measures that are derived from clinical observations, mainly the revised ALS Functional Rating Scale (ALSFRS-R) or long-term survival rates (Benatar et al., 2022). The recent example of the agent Tofersen underlines the importance of other outcome measures that were pivotal for its marketing authorization tailored to the minority of patients with ALS carrying a variant in the SOD1-gene. While the primary outcome measure of ALSFRS-R decline failed to reveal a significant effect in the respective phase 3 study, it was observed that Tofersen led to greater reductions in concentrations of SOD1 in cerebrospinal fluid (CSF), and of neurofilament light chains in plasma than placebo (Miller et al., 2022). This illustrates the potential of biomarkers to enhance the advancement of clinical trials. However, more research is necessary to identify and validate specific pharmacodynamic, prognostic, or predictive biomarkers in order to ensure that signals observed from these surrogates are indeed clinically meaningful (Kiernan et al., 2020).

Studying the central nervous system compartment CSF, neurofilaments are widely considered as promising biomarkers for various neurodegenerative and neuroinflammatory conditions, due to their neuronal specificity (Verber and Shaw, 2020; Abu-Rumeileh et al., 2022). Neurofilaments constitute part of the intermediate filament family, comprising light (NfL), middle and heavy chains of varying weights and are essential components of the axonal cytoskeleton (Gentil et al., 2015; Khalil et al., 2018; Hohmann and Dehghani, 2019). They are explicitly expressed during neuronal growth and maturation-, in large-myelinated neurons, which make them particularly interesting for research in motor neuron diseases (Bomont, 2021; Zecca et al., 2022). The neurofilament isoforms assemble and form compound-filaments which provide structural stability for the neurons. They are also involved in transport and docking of organelles (Yuan et al., 2017; Zucchi et al., 2020). The phosphorylation of neurofilament heavy chain tails occurs as post-translational modification and indicates the interaction with neighboring filaments that regulate their axonal transport rate (Gentil et al., 2015; Bomont, 2021). Amyotrophic lateral sclerosis is associated with significantly elevated CSF-levels of neurofilaments that are likely caused by the axonal damage, but may also be directly related to the pathophysiological process of this neurodegenerative disease (Khalil et al., 2018).

Studies comparing CSF levels of neurofilament light (NfL) vs. phosphorylated heavy chains (pNfH) have suggested that the two have different sensitivity and specificity for ALS (Poesen and Damme, 2018). Moreover, a former study by Menke et al. (2015) suggested that pNfH correlates better with clinical signs of lower motor neuron damage than NfL, that on the other side has been described to correlate with upper motor neuron dysfunction. During the progressing ALS disease, longitudinal studies revealed that pNfH CSF concentrations remain relatively stable, while some studies assessing NfL serially reported unstable levels (Lu et al., 2015; Steinacker et al., 2015; Poesen et al., 2017). This would qualify pNfH as the preferred candidate biomarker that may be assessed at any time during the symptomatic phase of the disease (Simonini et al., 2021; Dreger et al., 2022; Heckler and Venkataraman, 2022). In principle, recent studies supported that the level of CSF pNfH mirrors the rate of neuroaxonal breakdown as it correlated with survival (Steinacker et al., 2015; Simonini et al., 2021; Kläppe et al., 2024). Concerning the association with progression speed of the disease, the ALSFRS-R derived disease progression rate (DPR) was used as linear approximation of the speed of disease progression (Labra et al., 2016), but studying the association with CSF concentrations of pNfH yielded mixed results. Some authors described a significant correlation with the DPR in limited cohorts of patients with ALS (McCombe et al., 2015; Schaepdryver et al., 2018; Simonini et al., 2021; Benatar et al., 2024), while others failed to reveal such a correlation (Li et al., 2016).

A common reason for the controversial results of these previous studies may be the weakness of the clinical measure, as the DPR assumes a linear decline of ALSFRS-R sum scores in progressing disease. By contrast, previous large-scale observations showed that the rate of decline varies throughout the individual course of the disease and follows rather a curvilinear course (Proudfoot et al., 2016; Ramamoorthy et al., 2022). In addition, the calculation of a progression-rate based on a single score, is highly susceptible to the known intra-rater and inter-rater variability associated with ALSFRS-R scoring (Bakker et al., 2020).

**Figure 1:**
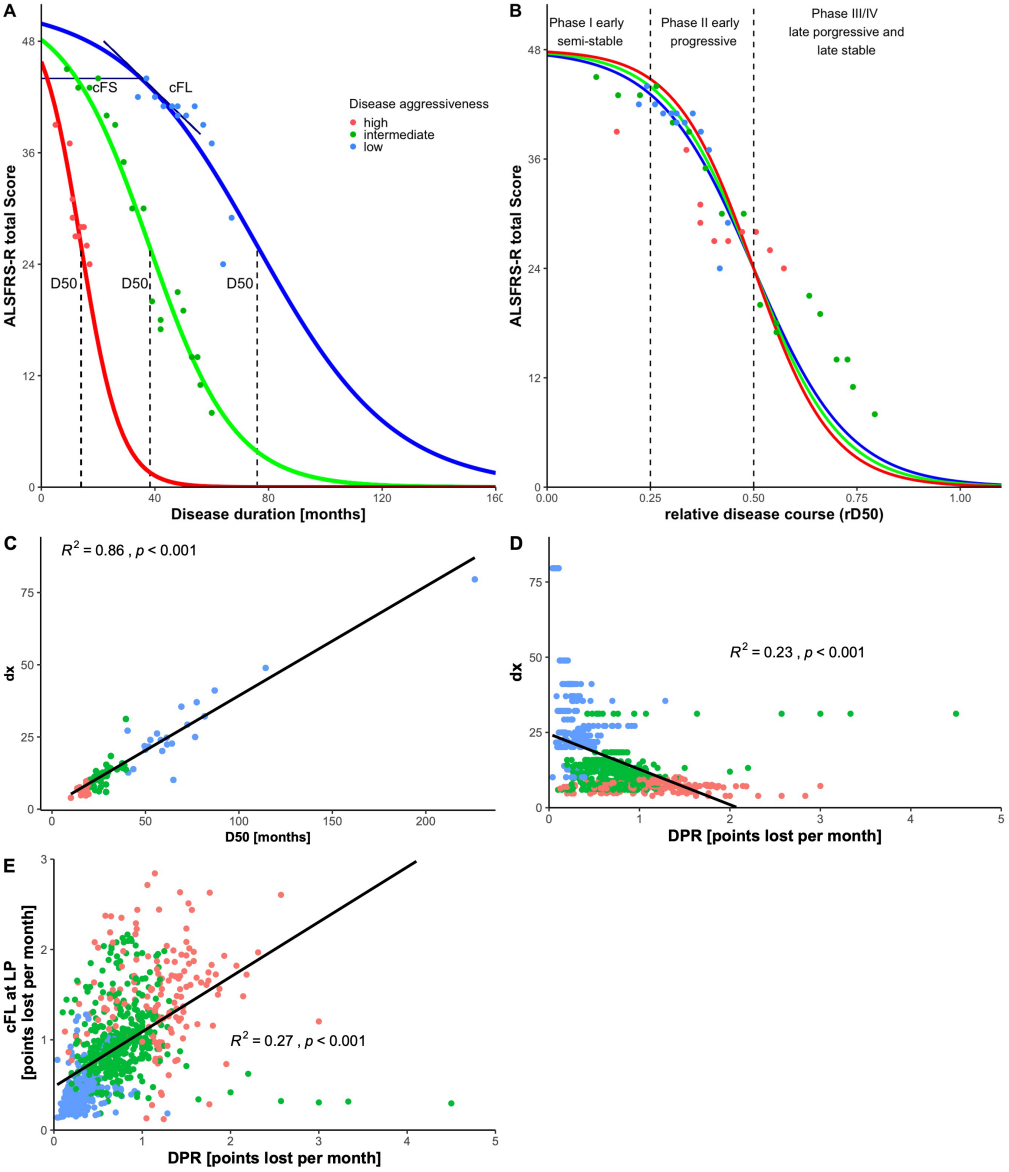
D50-modeling of disease progression in the ALS cohort (*n* = 108). **(A)** Based on consecutively assessed ALSFRS-R scores (dots), a sigmoidal functional decline curve is calculated. The value D50 depicts the individual time in months since symptom onset until halved functionality, indicating the overall disease aggressiveness of each individual patient. The curves represent three examples of patients with either high (in red), intermediate (in green), or low disease aggressiveness (in blue). Descriptors of local disease activity can be calculated for any given time point (here: day of lumbar puncture): (i) the calculated Functional Loss-rate (cFL) that measures the acute decay rate of points lost per month; (ii) the Calculated Functional State (cFS) that uses the same scale as the ALSFRS-R score. **(B)** Normalizing the D50-value onto 0.5 yields the relative D50 (rD50) which measures individual disease covered/accumulation, independent of disease aggressiveness and can be calculated for any given timepoint (here: day of lumbar puncture). Based on rD50, the disease course can be divided into distinct phases: (a) the early semi-stable Phase I (rD50 < 0.25), (b) the early progressive Phase II (0.25 ≤ rD50 < 0.50), and (c) the late progressive/stable Phases III/IV (rD50 ≥ 0.50). **(C)** Direct correlation of dx, the time constant of functional decline, and D50 is highly significant which explains why D50 alone can be used to describe the functional decline. **(D)** Illustrates the diversification of conservatively linear-approximated disease progression rates (DPR; points lost per month) per patient in relation to modeling parameters with only minor determination: DPR with dx (*R*^2^ = 0.23) and **(E)** DPR with cFL (*R*^2^ = 0.27).

The D50 model of ALS disease progression was developed in order to overcome such limitations of traditional clinical metrics. It has already proven to facilitate robust correlations with values originating from various biomarker signals (Gaur et al., 2023; Magen et al., 2021; Steinbach et al., 2021). Briefly, the model characterizes the progressive decline in motoneuronal capacity from full health to functional loss (Figure 1). It quantifies overall disease aggressiveness as the time taken to reach halved functionality (parameter D50) and further enables the calculation of individual disease covered/accumulation (e.g., in distinct phases) and of acute descriptors of local disease activity. Strengths of the D50 model in comparison to traditional disease metrics are that it takes into account the individual clinical course as a whole and reflects its typically curvilinear decline of disability more appropriate (Ramamoorthy et al., 2022). It thus enables unbiased comparisons of patients with vastly differing time courses of the disease in cross-sectional cohorts (so-called pseudo-longitudinal approach). Furthermore, it reduces noise inherent with ALSFRS-R assessment mentioned before, as it incorporates multiple serial measurement time-points per patient instead of a singular observation.

In this study, we applied the D50 model to a heterogeneous cohort of patients with ALS to investigate if CSF pNfH concentrations would correlate with measures of disease progression speed independent of disease accumulation. We additionally hypothesized that CSF pNfH levels would capture patients’ overall disease aggressiveness (as measured by D50) better than parameters measuring local rate of progression. If confirmed, this would underscore the potential of pNfH as meaningful outcome measure thus reflecting overall disease aggressiveness of the symptomatic stages of the ALS disease, independent of the time of assessment (i.e., lumbar puncture).

## 2 Materials and methods

### 2.1 Participants

**Figure 2:**
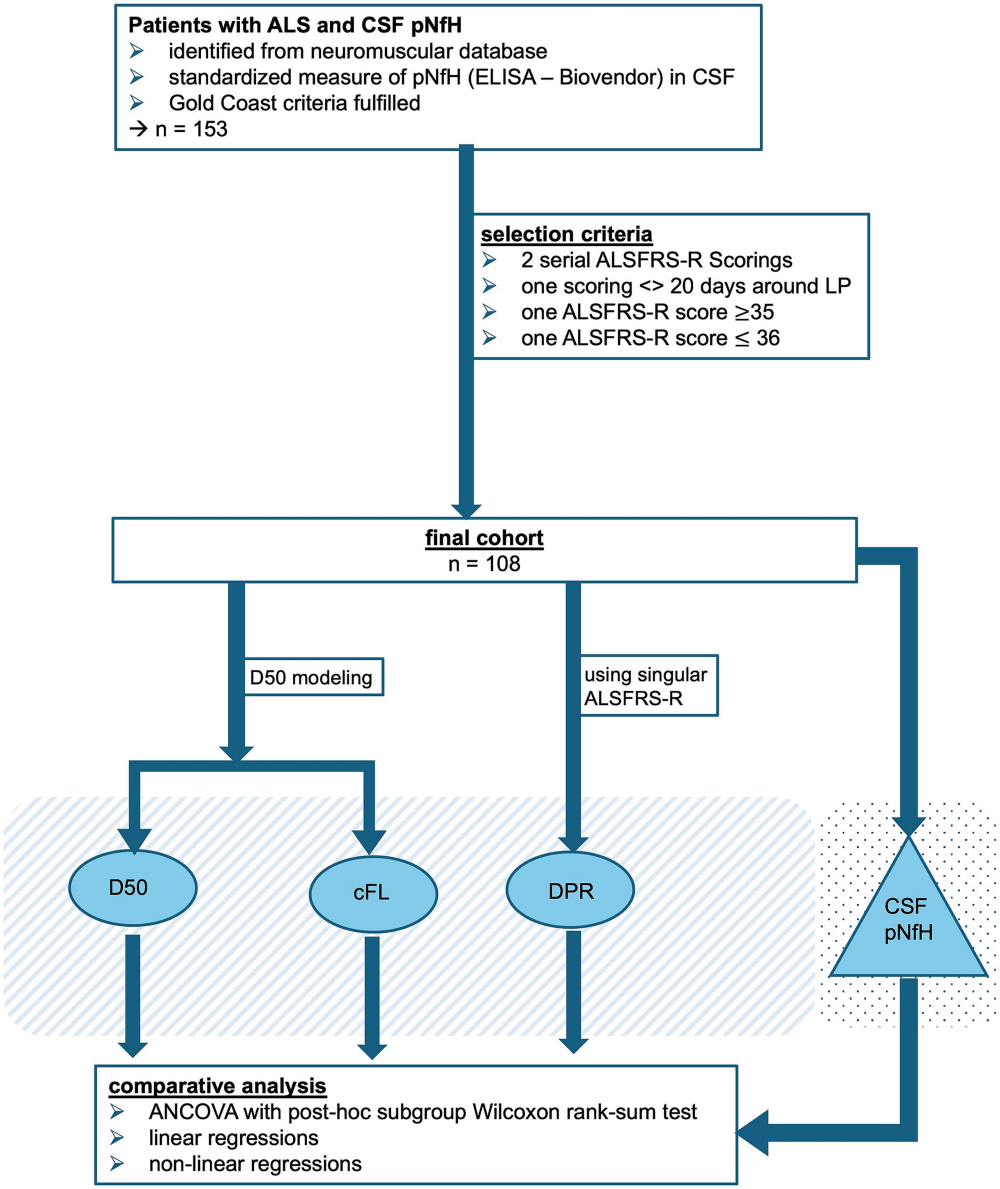
Study flow of study procedures. Identification and selection of the study cohort is given, resulting in the final cohort of 108 patients with ALS and ELISA quantified concentrations of pNfH in CSF. Three different measures of disease progression speed were calculated: (i) D50 = overall disease aggressiveness, (ii) the calculated functional loss-rate (cFL) = locally derived parameter of progression speed and (c) the disease progression-rate (DPR) as linear approximation of ALSFRS-R points lost per month since symptom onset. Each of the progression speed parameters was comparatively analyzed in relation to CSF pNfH levels via analyses of covariance, linear and non-linear regressions.

All participants were recruited from the Neuromuscular Center at Jena University Hospital (Germany) between the years 2013 and 2020 and written informed consent was obtained prior to study initiation. All procedures were approved by the local ethics committee (Nr. 3,633–11/12) and were conducted in accordance with the Declaration of Helsinki and its later amendments. A total of 153 individuals diagnosed with motor neuron disease and available CSF samples were identified from the local specialized neuromuscular disease database (Steinbach et al., 2020). We only included patients who fulfilled the Gold Coast criteria for the diagnosis of ALS as assessed by a specialized physician (Shefner et al., 2020). To allow stringent D50 modeling and comparison with the traditional DPR, we only included individuals who met all of the following criteria: (a) ≥2 recorded ALSFRS-R scores available, (b) at least one score taken within 20 days before/after CSF sampling, (c) at least one score ≥ 35 and (d) at least one score ≤ 36. This led to the exclusion of 57 individuals and the final cohort included 108 patients with ALS (see also Figure 2 for an overview of the study procedures). According to patients’ reports of the first site of motoric function loss, the clinical onset was allocated to the bulbar or spinal (i.e., limbs) region. Furthermore, clinical phenotypes were classified as either classic, bulbar, pyramidal, flail arm, flail leg, respiratory or pure lower motor neuron as described by Chiò et al. (2011). The presence of comorbid frontotemporal dementia was assessed according to the Strong criteria (Strong et al., 2009, 2017).

### 2.2 CSF sampling and analysis

Cerebrospinal fluid (CSF) samples were obtained via lumbar puncture at the Department of Neurology, Jena University Hospital following an in-house standard operating procedure. Accordingly, the CSF samples were subjected to centrifugation (5,000 × *g*, 10 min, 4°C), aliquoting, and cryopreservation at −80°C within 2 h after lumbar puncture until further use. The pNfH concentrations were determined using a commercially validated ELISA kit (Biovendor International, RD191138300R); assays were performed in accordance with manufacturer instructions at one of two European laboratories in Germany (*n* = 56) and Belgium (*n* = 52) that are accredited for these analyses. All samples were analyzed in duplicate, and the intra- and inter-assay coefficients of variation were both ≤10% and ≤20%, respectively.

### 2.3 The D50 disease progression model

The D50 Disease Progression Model provides a detailed framework to interpret any biomarker signal as it provides quantitative measures of disease aggressiveness that are distinct from parameters of disease accumulation (Poesen et al., 2017; Steinbach et al., 2020). Briefly, the model calculates an individual sigmoidal state transition from functional health to motor function loss, based on all available ALSFRS-R scores longitudinally collected on a regular basis for the individual patient (Figure 1A). The resulting sigmoidal curve can be characterized by two parameters: (a) D50, that represents the time in months since symptom onset until the time point of halved functionality, and (b) dx, that represents the time constant of functional decline, i.e., the steepness of the curve. Repeated observations showed that both parameters D50 and dx exhibit a direct intercorrelation in different cohorts (Poesen et al., 2017; Prell et al., 2019; Steinbach et al., 2020) as also confirmed for the patient sample of this study (*R*^2^ = 0.86, *p* < 0.001; Figure 1C). Therefore, the D50 value alone can be employed to describe the curve and thus provides a discrete descriptor of the overall disease aggressiveness of an individual patient. The patients of this study were thus categorized into three subgroups of (a) high (D50 < 20 months), (b) intermediate (20 ≤ D50 < 40 months), and (c) low (D50 ≥ 40 months) disease aggressiveness. The relative D50 (rD50) parameter is obtained by normalizing the patient’s real-time sigmoidal disease trajectory to D50. This results in an open-ended reference scale, with 0 representing symptom onset and 0.5 representing the time point at which functionality is halved (Figure 1B). The rD50 provides an individualized quantification of disease accumulation, independent of disease aggressiveness, that can be calculated for any given time-point and was calculated in this cohort for the day of lumbar puncture. Based on the observations of rD50 modeling, patients can be classified into one of three phases of similar disease progression patterns that they pass during the course of their disease: (a) the early semi-stable phase I (0 ≤ rD50 < 0.25), (b) the early progressive phase II (0.25 ≤ rD50 < 0.5), and (c) the late progressive and stable phase III/IV (0.5 ≤ rD50) (Figure 1B). Besides, the model also provides the opportunity to calculate descriptors of local disease activity, for any given time point, namely the calculated Functional State (cFS) and the calculated Functional Loss-rate (cFL). The latter was calculated in this study for the individual time of lumbar puncture as it provides a measure of the acute decay rate of points lost per month, thus the local steepness of the sigmoidal curve (Figure 1A). Patients were categorized into subgroups of (a) fast (cFL > 1), (b) intermediate (0.5 > cFL ≤ 1), and (c) slow (cFL ≤ 0.5) local loss-rate. For comparison purposes, the traditional DPR was also assessed, as linearly approximated based on the ALSFRS-R nearby the day of lumbar puncture and measured in points lost per month since symptom onset. Similar borders were applied for categorization of the cohort, i.e., (a) fast (DPR > 1), (b) intermediate (0.5 > DPR ≤ 1), and (c) slow (DPR ≤ 0.5).

### 2.4 Statistical analysis

The statistical analyses and graphical representation of the data were conducted using the R programming language and the RStudio open-source software program (Version 2023.06.1, Posit Software, PBC, Boston, MA, United States) used on macOS Sequoia 15. Normality was assessed using the Shapiro–Wilk test with a log-10 transformation applied to the following continuous variables to allow the application of parametric tests: pNfH concentration, D50, cFL and DPR.

To assess the differences in log[pNfH]-concentrations across distinct D50-derived ALS subgroups, namely low, intermediate, and high aggressiveness, a one-way analysis of covariance (ANCOVA) was conducted, employing the following covariates: age at LP, presence of FTD, sex, laboratory of pNfH measurement, clinical onset region and rD50-derived disease phase. In addition, the same methodology was applied using subgroups based on the cFL and DPR instead of the D50 subgroups. The ANCOVAs were followed by *post hoc* analyses with Wilcoxon-Rank-Sum-Testing.

Linear regression models were constructed for each of these three disease progression speed variables with log[pNfH] as dependent variable. For more in-depth analyses of these potential associations, non-linear regression models were applied using the *goodness-of-fit* (*chemdeg* package; Version 0.1.4) and *gls-nls* (Version 1.3.2) sub-programs in R. The non-linear regression in general gives information about the model used for correlation with data, with a mathematical function as non-linear (bent) output. Subsequently, a *goodness-of-fit* analysis was conducted, in order to assess how well the fitted curve predicts the real data. The Akaike Information Criterion (AIC) was employed to compare the relative merits of alternative models in order to identify the optimal fit for the data in question. A lower AIC-value indicates a better prediction. Additionally, the Root-Mean-Square Error (RMSE) was employed as a non-negative measure, offering a valuable metric for comparing forecasting errors across diverse predictive models. The RMSE provides an absolute measure of fit, where a lower RMSE value indicates a superior fit of the model, i.e., that the prediction is closer to the real values.

## 3 Results

### 3.1 Cohort of patients with ALS

**Figure 3:**
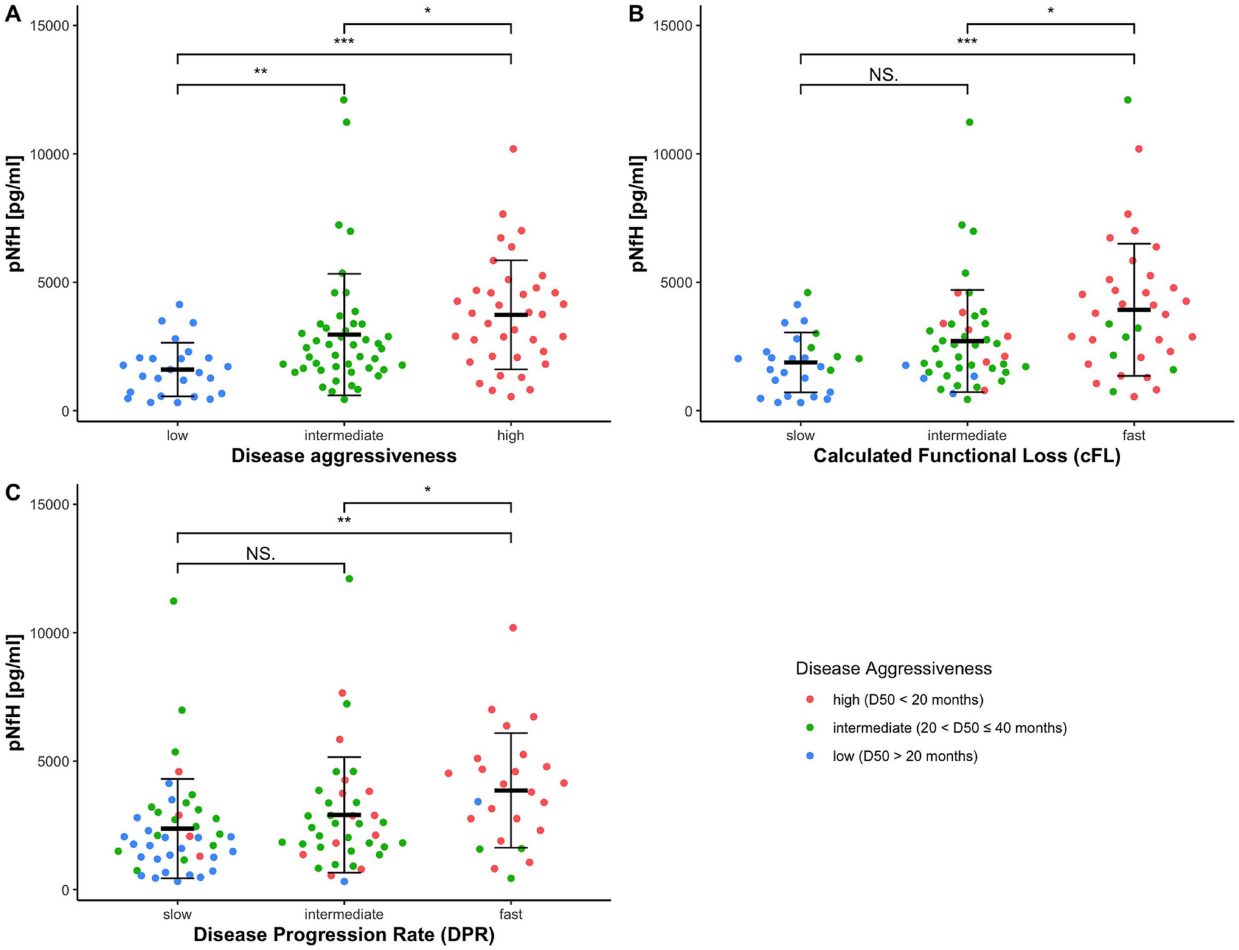
Cerebrospinal fluid (CSF) pNfH concentrations in pg/ml compared between different ALS subgroups, each stratified by a disease progression speed variable show that **(A)** D50-derived subgroups are best discriminative. **(B)** cFL-derived subgroups are less discriminative and **(C)** DPR-derived subgroups even worse concerning pNfH levels. With significance levels from *post hoc* Wilcoxon-Rank-Sum-Testing: * = *p* < 0.05, ** = *p* < 0.01, *** = *p* < 0.001; NS, no significance; stratified into D50-derived disease aggressiveness with low (blue; D50 > 40 months), intermediate (green; 20 < D50 ≤ 40 months) and high aggressiveness (red; D50 < 20 months).

The demographic and clinical data of the participants, stratified by D50-derived disease aggressiveness subgroups, are presented in Table 1. No significant inter-subgroup differences were observed concerning sex or age. Notably, disease accumulation at the day of sampling, as measured via relative D50 (rD50), did not significantly differ between the three aggressiveness subgroups. Accordingly, the distribution of rD50-derived phases did not differ either, thus confirming independence of disease accumulation and aggressiveness in this cohort. Concerning the parameters cFL and DPR, we also found significant differences across the aggressiveness subgroups, indicating partial but not entire overlapping of these subgroups (see also Figure 3).

**Table 1:** Demographic and clinical data for Patients with ALS (*n* = 108).

Disease aggressiveness				*p*
	Low (D50 ≥ 40)	Intermediate (20 ≤ D50 < 40)	High (D50 < 20)	
*n*	25	46	37	–
Phosphorylated Neurofilament Heavy chain (pNfH) measurement				
pNfH [pg/mL]^§^	1,482 (666–2,053)	2,432 (1,651–3,332)	3,747.5 (2,115.2–4,683.0)	<0.001*
Laboratory: Germany/Belgium	16/9	28/18	22/15	0.13
Demographics				
Age at lumbar puncture^§^	64.67 (58.17–72.17)	65.17 (59.02–71.85)	68.42 (59.58–71.92)	0.4821
Sex [n]: male/female	15/10	25/21	22/15	0.857
D50 disease progression model parameters				
D50^§^	64.17 (52.60–81.67)	28.56 (23.79–31.02)	15.475 (12.21–18.43)	<0.001*
rD50^§^	0.223 (0.155–0.302)	0.232 (0.025–0.275)	0.251 (0.125–0.376)	0.8484
Phase [n]				0.5427
I (rD50 < 0.25)	16 (64%)	27 (58.7%)	18 (48.6%)	
II (0.25 ≤ rD50 < 0.5)	9 (36%)	18 (39.1%)	19 (51.4%)	
III/IV (rD50 ≥ 0.5)	0	1 (2.2%)	0	
Traditional disease metrics				
ALSFRS-R at lumbar puncture^§^	42 (39–44)	41 (39–44)	39 (36–42)	0.0849
Disease duration at lumbar puncture^§^[months]	29 (17–46)	12.50 (9–16)	7 (6–10)	<0.001*
cFL^§^	0.31 (0.18–0.43)	0.77 (0.64–0.87)	1.37 (1.12–2.17)	<0.001*
DPR^§^	0.17 (0.12–0.33)	0.58 (0.47–0.72)	1.2 (0.75–1.8)	<0.001*
Onset region of first symptoms [n]				0.0106*
Bulbar	4 (16%)	18 (39.1%)	20 (54.1%)	
Spinal	21 (84)%	28 (60.9%)	17 (45.9%)	
ALS Phenotype (Chiò et al., 2011) [*n*]				<0.001*
Classic	3 (52%)	31 (67.4%)	20 (54.1%)	
Flail arm	4 (16%)	0	0	
Bulbar	3 (12%)	14 (30.4%)	15 (40.5%)	
Flail leg	2 (8%)	0	0	
Pyramidal	2 (8%)	1 (2.2%)	2 (5.4%)	
Pure LMN	1 (4%)	0	0	
Presence of FTD [*n*]: yes/no	1/24	0/46	0/37	0.1872
Riluzole treatment [*n*]: yes/no	18/7	41/5	34/3	0.06245

Continuous variables with normal distribution are expressed as mean with standard deviation. Categorical variables are expressed as number and percentage. For the comparison of demographic and clinical variables among the three aggressiveness subgroups, analyses of variance, Kruskal–Wallis tests or Pearson – *χ*^2^ tests were applied where appropriate.

^§Non-parametric nominal variables, represented as median and interquartile range.^

^*^Statistical significance at *p* < 0.05.

ALS, amyotrophic lateral sclerosis; ALSFRS-R, Revised ALS Functional Rating Scale; cFL, calculated Functional Loss-rate; FTD, frontotemporal dementia; pNfH, phosphorylated neurofilament heavy chain; DPR, ALSFRS-R-based Disease Progression Rate; rD50, relative D50; LMN, lower motor neuron.

### 3.2 CSF pNfH levels reflect disease aggressiveness in ALS

The ANCOVA demonstrated a highly statistically significant effect for CSF log[pNfH] concentrations across the three disease aggressiveness subgroups (*p* < 0.001, Table 2). In contrast, the ANCOVAs applying either the cFL or DPR as grouping variables, demonstrated higher but still significant *p*-values for this factor of 0.005 or 0.006, respectively, (>0.001) but notably also lower *F*-values (D50: 10.43; cFL: 5.58; DPR: 5.3). In accordance, the *post hoc* analyses indicated statistical significance only for the pairwise comparisons of the subgroups from both edges of the grouping, i.e., fast-vs.-slow cFL/DPR (*p* < 0.01) (Figure 3). It is of particular importance to note that the disease phase did not exert a significant effect on log[pNfH] concentrations in any of the ANCOVAs conducted. Accordingly, the linear regression of log[pNfH] and rD50 did not reveal a statistically significant correlation (Supplementary Figure 1). This indicates independence of pNfH levels from disease accumulation. Notably, almost all other covariates applied in the ANCOVAs did also not show any significant influence. Only the factor age at sampling was nearer to the significance threshold for the D50/cFL subgrouping (*p* = 0.09/0.08, Table 2), but decided the threshold for the ANCOVA applying DPR subgrouping thus indicating a significant age influence on log[pNfH] levels (*p* = 0.02, Table 2).

**Table 2:** Analysis of covariance (ANCOVA).

Tests of between-subjects effects									
Dependent variable: Log[pNfH]	D50			cFL			DPR		
Factor	df	*F*	*p*	df	*F*	*p*	df	*F*	*p*
D50/cFL/DPR – subgroups	2	**10.4300**	0.000078*	2	**5.5841**	0.005051*	2	**5.3046**	0.006495*
rD50-derived disease phase (I/II/III and IV)	2	1.0482	0.3722	2	0.4327	0.650009	2	1.8456	0.163373
Age at LP	1	2.8603	0.09397	1	3.0571	0.083517	1	**5.5861**	0.020077*
Laboratory (Germany/Belgium)	1	1.8809	0.17337	1	1.9764	0.162928	1	3.0878	0.082005
Sex (male/female)	1	2.0517	0.15521	1	1.4934	0.224618	1	3.0543	0.083656
FTD (yes/no)	1	0.9324	0.33662	1	1.1623	0.283632	1	1.7517	0.188745
Onset region of first symptoms (bulbar/spinal)	1	0.4455	0.50603	1	0.1363	0.712762	1	0.0028	0.958209

Analysis of covariance (ANCOVA) results of the filtered cohort applying D50, cFL or DPR subgrouping, respectively.

^*^Statistical significance at *p* < 0.05, additionally highlighted in bold.

cFL, calculated Functional Loss-rate; FTD, frontotemporal dementia; LP, lumbar puncture; pNfH, phosphorylated Neurofilament Heavy chain; DPR, ALSFRS-R-based Disease Progression Rate; rD50, relative disease covered in disease phase.

The pairwise comparisons of pNfH concentrations in-between the D50-derived disease aggressiveness subgroups revealed the most conclusive differences, i.e., *p* < 0.05 for high vs. intermediate subgroup, *p* < 0.01 concerning the low and intermediate subgroup, and *p* < 0.001 comparing the low with the high disease aggressiveness subgroup (Figure 3A). The subgroups defined by cFL were less discriminative concerning pairwise comparisons of pNfH-concentrations that were not significantly different between the slow and intermediate subgroup (Figure 3B). In DPR-defined subgroups pNfH performed even less discriminative especially for the extreme subgroup comparisons (slow vs. fast, *p* < 0.01) (Figure 3C).

**Figure 4:**
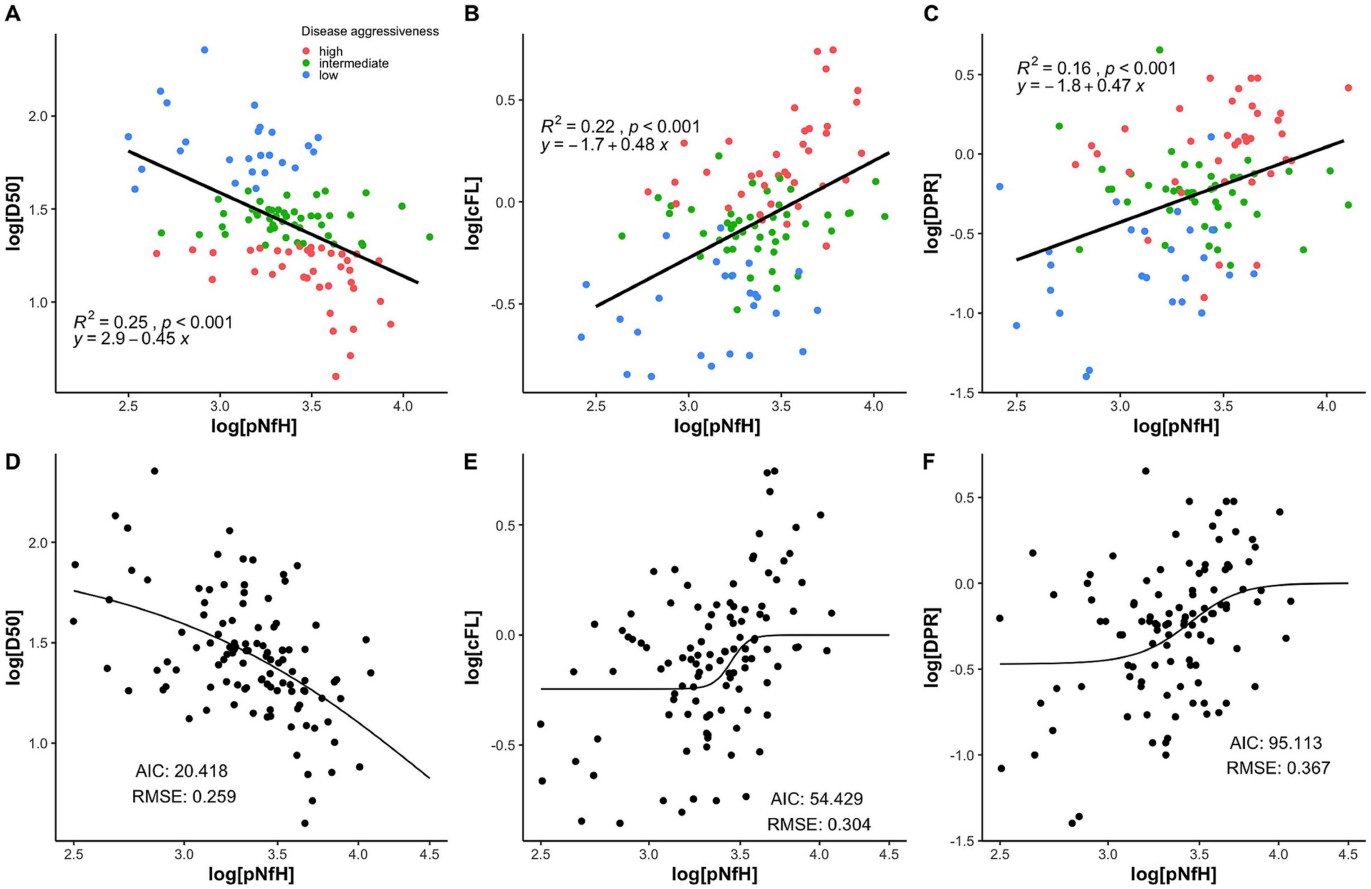
**(A–C)** Linear regressions of disease progression speed paramters with CSF pNfH showed the highest *R*^2^ of 0.25 and the lowest *p*-value for D50 in comparison to cFL and DPR. **(D–F)** Non-linear regressions also showed superiority for D50 with the best goodness-of-fit meassures (Akaike Information Criterion = 20.413 and Root Mean Square Error = 0.259).

The three linear regression analyses, each applying one of the parameters assessing disease progression speed, demonstrated all statistically significant correlations with log[pNfH] (*p* < 0.001; Figures 4A–C). However, the linear model using log[D50] exhibited the highest coefficient of determination (*R*^2^) of 0.25 and the lowest *p*-value (Figure 4A) while in comparison log[DPR] performed worst (*R*^2^ = 0.16; Figure 4C). As the association between CSF pNfH-concentrations and ALS progression speed parameters is apparently not linear, we additionally applied a non-linear regression model to further examine the correlation. This exhibited markedly disparate plots of correlation-curves with log[pNfH] across all three variables (Figures 4D–F). Log[pNfH] revealed a more shaped curve (Figure 4D), whereas the non-linear regression-curves for log[cFL] and log[DPR] demonstrated a clinched sigmoidal shape (Figures 4E,F). In accordance, the non-linear regression model applying log[D50] exhibited the most optimal *goodness-of-fit* measures, with an AIC of 20.418 and an RMSE of 0.259.

## 4 Discussion

In the present study, the principal aim was to examine which clinical parameter assessing disease progression speed is best correlated with pNfH-concentration in the CSF of patients with ALS. In principle, pNfH-levels were able to discriminate in-between subgroups as defined by all three disease speed parameters (D50, cFL, DPR) respectively (Table 2). However, *post hoc* analyses showed, that only the D50-parameter was able to significantly discriminate pairwise across all three aggressiveness subgroups, while this approach failed for the slowest subgrouping applying the other parameters (fast-vs.-slow cFL/DPR; Figure 3). The regression models (linear and non-linear) did also reveal superiority of the parameter D50 while DPR and cFL performed worse in correlation with pNfH-levels (Figure 4).

Some studies with limited cohorts investigated potential associations between pNfH levels in CSF and disease progression in ALS so far, applying the singular disease metric of the linearly approximated DPR (Steinacker et al., 2015, 2018; Li et al., 2016; Poesen et al., 2017; Schaepdryver et al., 2018; Thompson et al., 2019; Behzadi et al., 2021). Other studies reported correlations between CSF pNfH and progressiveness applying linear mixed models with more locally assessed disease progression rates (Huang et al., 2020) and another measured the time to generalization of clinical symptoms (Li et al., 2016). It was mentioned before that the high inter-patient variability observed in ALS disease progression necessitates the usage of a framework that can holistically interpret the progression of the disease and putative biomarkers. Therefore, the D50 model was applied in this study enabling a comprehensive analysis of a well-defined cross-sectional dataset. An important advantage of this progression-modeling is that it provides quantifications of disease aggressiveness (D50) separately from disease accumulation (rD50, phase). According to this pseudo-longitudinal approach, we were able to show that CSF pNfH levels remain stable throughout the symptomatic disease, because we did not find a significant effect of disease phase nor a direct correlation between rD50 and pNfH (Supplementary Figure 1). This is in line with previous real-longitudinal studies that also reported stability of pNfH-levels, however, owing to the invasive nature of CSF collection, only a limited number of patients had been examined via serial lumbar punctures before (Steinacker et al., 2015; Gendron et al., 2017; Poesen et al., 2017; Huang et al., 2020). The pseudo-longitudinal approach applied in this study further confirmed that CSF pNfH is strongly associated with patient’s disease aggressiveness, independent of how far the disease has progressed at the time of sampling. This would promote a broader inclusion of patients in therapeutic studies using pNfH as prognostic marker. However, it should be noted that the patients included here were in disease phases 1 or 2. Otherwise, biomarkers for patients who have already progressed to disease phase 3/4 are unlikely to be useful for upcoming interventional studies (Benatar et al., 2022). Notably, the only additional parameter that showed borderline influence on pNfH levels was age. Increasing neurofilament-levels with aging are known from former studies in cohorts of healthy individuals (Khalil et al., 2020; Witzel et al., 2024). However, the results of this study suggest that in the context of ALS the influence of age is of minor importance. It is remarkable that although ELISAs were performed in two different laboratories (Belgium and Germany) we found no significant impact of the factor analyzing laboratory on pNfH levels. A previous study testing the same samples in different laboratories in a “round-robin” comparison revealed low coefficients of variation for pNfH-CSF ELISAs, while those for CSF NfL tended to be relatively less reliably replicable (Gray et al., 2020). Although this should be interpreted with caution it might be that CSF pNfH is the better candidate if analyzed in inter-laboratory conditions, perhaps due to higher pre-analytical stability. This opens perspectives to use our approach in multi-center trials, if standards for biofluid collection and neurochemical laboratory analysis are considered (Oeckl et al., 2016; Benatar et al., 2020).

After all, it could be demonstrated that pNfH levels in CSF are more increased in ALS patients with higher disease aggressiveness, even after adjustment for interlaboratory variations, age, sex, presence of FTD, ALS onset region and disease phase. Applying grouping based on cFL and DPR, pNfH-levels were also partly discriminative in-between subgroups, but most noteworthy did not reveal any significant differences between slow and intermediate subgroups. This superiority of D50 in relation to pNfH is remarkable as this parameter is a time constant describing the overall course of the whole disease contrary to local, time-dependent decay-rates such as the cFL. This supports the concept that pNfH measured at any time during the disease may serve as outcome measure of an individual overall disease trajectory. As such, our data suggest that CSF pNfH might be a suitable biomarker for therapeutic responses, but further studies are needed to assess its potential as outcome measure in ALS, as replications in independent cohorts are necessary.

Therefore, one important advantage of the D50-model framework is that it can be applied retrospectively provided that serial ALSFRS-R assessments are available per patient. This opens perspectives for multi-center analyses including highly-frequent clinical (self-)assessments of patients from different sources (Maier et al., 2022; Meyer et al., 2023). However, thorough review and regular follow-up by personal trained in neuromuscular disease is necessary as is a specialized preferably milestone-based documentation of such data (Steinbach et al., 2020). Although the strong association between CSF pNfH and D50 demonstrated in this study is promising, it should be noted that it relies on observations on a group-level. For the development of a prognostic biomarker that is applicable on an individualized level it is likely that a combination of surrogates from different sources is needed, such as blood biomarkers or imaging (Dreger et al., 2022; Staffaroni et al., 2022).

Former comparative studies measuring pNfH in CSF as well as in blood demonstrated good accordance, but still the latter is often not analyzed in ALS cohorts on a regular basis (Boylan et al., 2013; Schaepdryver et al., 2018). A recent study suggested that blood-levels of pNfH may be less valuable than NfL in association with disease progression, however this could be attributed to inaccuracies of pNfH blood-assays that are still technically challenging (Sturmey and Malaspina, 2022; Benatar et al., 2024). Moreover, although blood-based biomarkers are easier assessable, this becomes less important in the context of repeatedly intrathecal-administered therapeutics such as Tofersen or other genetically-based agents currently under consideration for ALS (Boros et al., 2022; Wiesenfarth et al., 2024). In line with this, recent real-world studies of treatment with Tofersen in SOD1 ALS patients showed a marked decline of serum NfL as well as CSF pNfH mirroring clinical disease stabilization (Wiesenfarth et al., 2024; Smith et al., 2025).

It is important to note, that we were not able to provide similar comparative analyses of NfL levels (serum or CSF) in combination with the clinical parameters, mainly due to the retrospective nature of the study. A former analysis studying another cohort of patients with CSF NfL levels revealed that there is a strong correlation with D50, independent of rD50 (Dreger et al., 2021). In addition, since measurements of neurofilament blood-concentrations (most of all NfL) become increasingly available, this opens new perspectives for future large comparative studies for which the D50 model provides a suitable analysis framework.

Regarding the regression models across the different disease progression speed parameters, it is important to note that the non-linear regressions were most informative, again demonstrating a superiority of the parameter D50. This implies a recommendation for future studies to consider non-linear relations between potential biomarkers and clinical parameters.

This study is not without limitations. This is a monocentric study and replication of the results examining independent cohorts is necessary. In addition, comparative studies are needed, studying also NfL in CSF as well as serum concentrations of both neurofilaments in relation to the clinical parameters. In addition, verification through real longitudinal data is necessary to confirm if pNfH levels are stable throughout the disease course. Such future studies will require well-designed multi-center initiatives (Meyer et al., 2024) with implemented standard procedures of biofluid collection and laboratory analysis (Oeckl et al., 2016; Benatar et al., 2020). Furthermore, genetic profiles of the patients with ALS included were not available due to the retrospective nature of the study, which is why we could not examine possible implications of genetic subtypes on pNfH levels.

In conclusion, we were able to demonstrate that pNfH is a surrogate of overall ALS disease aggressiveness as quantified via D50 in a multi-variate and unbiased approach. The modeling thereby reduces noise associated with ALSFRS-R assessment and most important accounts for the non-linear clinical progression during the course of the disease (Proudfoot et al., 2016; Ramamoorthy et al., 2022). Future, independent multivariate studies are needed incorporating different analyte candidates with the principal aim of a composite outcome-measure and/or individually applicable prognostic biomarker, as urgently needed in the advent of precision therapy for ALS (Ashhurst et al., 2022).

## Data Availability

The raw data supporting the conclusions of this article will be made available by the authors, without undue reservation.
