# Supplementary figures and images for: Phosphorylated neurofilament heavy chain (pNfH) concentration in cerebrospinal fluid predicts overall disease aggressiveness (D50) in amyotrophic lateral sclerosis
Source: Front Neurosci. 2025 Mar 12;19:1536818. doi: 10.3389/fnins.2025.1536818 (PMC11936903; doi:10.3389/fnins.2025.1536818)

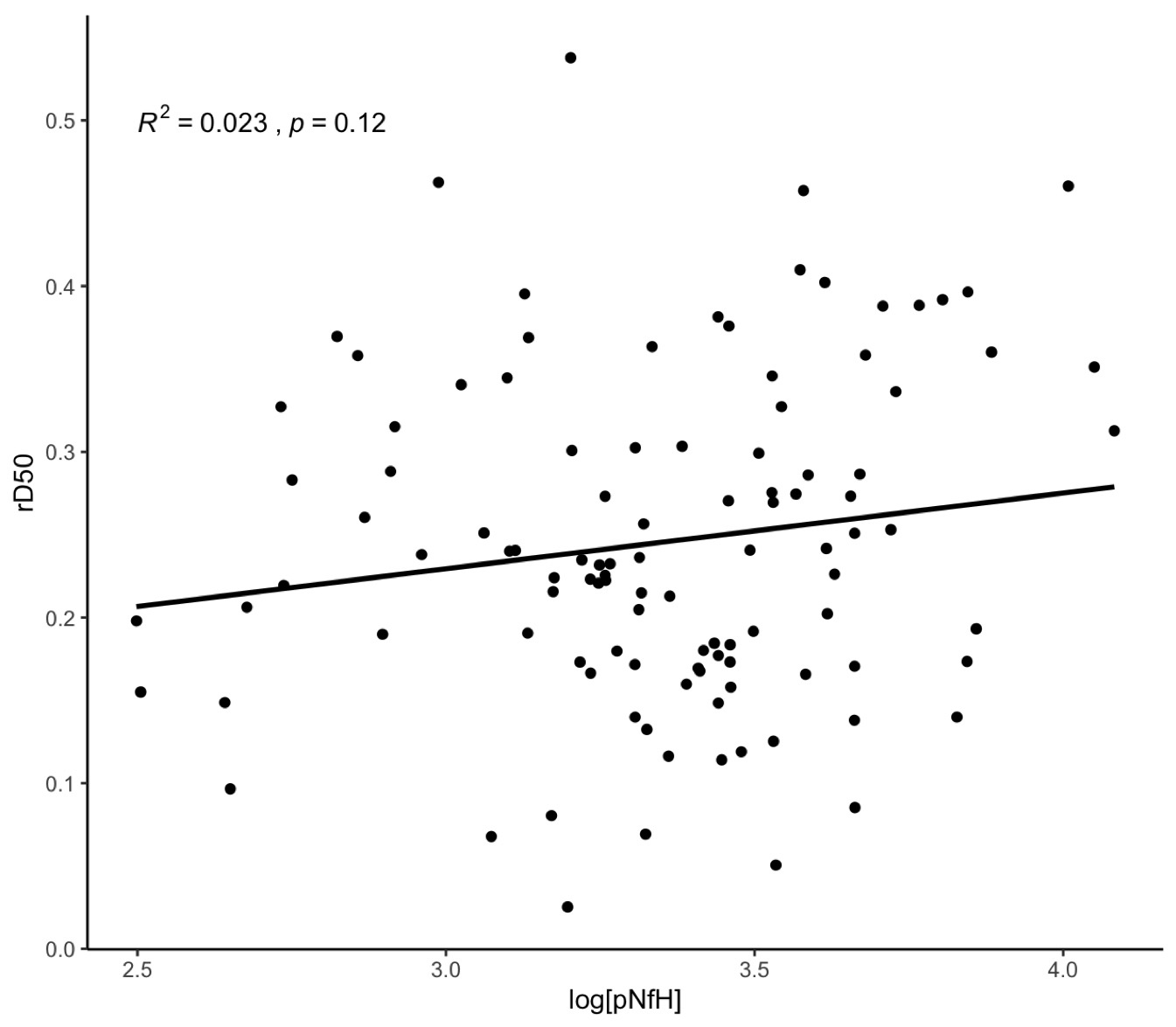

Supplement: Supplementary file 1 [file Image_1.JPEG]
